# Exploring the Discontinuous Usage Behavior of Digital Cognitive Training Among Older Adults With Mild Cognitive Impairment and Their Family Members: Qualitative Study Using the Extended Model of IT Continuance

**DOI:** 10.2196/66393

**Published:** 2025-03-25

**Authors:** Shangyang Zhang, Min Wu, Ruini Sun, Changjie Cui, Ziqing Zhang, Jing Liao, Ni Gong

**Affiliations:** 1 School of Nursing Jinan University Guangzhou China; 2 Department of Medical Statistics and Epidemiology School of Public Health Sun Yat-sen University Guangzhou China; 3 School of Sociology and Anthropology Sun Yat-sen University Guangzhou China; 4 School of Nursing Capital Medical University Beijing China; 5 Global Health Institute School of Public Health, Instiute of State Governance Sun Yat-sen University Guangzhou China

**Keywords:** digital cognitive training, discontinuous usage behavior, acceptance, mild cognitive impairment, qualitative study

## Abstract

**Background:**

Digital cognitive training (DCT) has been found to be more effective than traditional paper-and-pencil training in enhancing overall cognitive function. However, a significant barrier to its long-term implementation is that older adults with mild cognitive impairment (MCI) do not continue to use it or even show a dropoff in usage after the initial engagement. Such short-term engagement may limit the potential benefits of DCT, as sustained use is required to achieve more pronounced cognitive improvements. Exploring the reasons for the shift in discontinuous usage behavior is crucial for promoting successful DCT implementation and maximizing its positive effects.

**Objective:**

This study aimed to explore the intrinsic reasons for the transition from initial acceptance to discontinuous usage behavior among older adults with MCI throughout the DCT process, by employing the extended model of IT continuance (ECM-ITC).

**Methods:**

We employed a qualitative research methodology and conducted 38 semistructured interviews before and after the use of DCT (3 times per week over 1 month, with each session lasting 30 minutes) with 19 older adults with MCI (aged 60 years or older) and 4 family members between January and March 2024. Thematic analysis and deductive framework analysis were used to identify the reasons for the discontinuous usage of DCT, with mapping to the ECM-ITC.

**Results:**

Most participants failed to complete the standard dosage of DCT. Data analysis revealed the reasons for the shift to discontinuous usage. Despite their need to improve cognitive function, participants found the cognitive training confusing and discovered that DCT did not align with their preferred method of training upon actual use. The disparity between their vague expectations and reality, combined with the contradiction between the “delayed gratification” of DCT and their desire for “immediate gratification,” made it difficult for them to discern the usefulness of DCT. Participants also viewed DCT as an additional financial burden and tended to avoid training under family pressure. They relied on motivational measures, which further weakened their intention to continue DCT, ultimately leading to the inability to develop continuous usage behavior.

**Conclusions:**

Continuous usage behavior differs from initial acceptance as it evolves dynamically with user experience over time. To encourage older adults with MCI to persistently engage with DCT, it is essential to not only thoroughly consider their genuine preferences and the potential disruptions DCT may bring to their lives but also bridge the gap between expectations and actual experiences. While ensuring that older adults receive appropriate external incentives and encouragement, it is equally important to foster their intrinsic motivation, thereby gradually cultivating the habit of sustained DCT usage.

## Introduction

Cognitive dysfunction has become a major challenge in global health and social care, with progression to dementia being difficult to reverse [1,2] and imposing substantial disease and economic burdens on individuals, families, and society [3,4]. Mild cognitive impairment (MCI) is a transitional state between healthy cognition and early dementia [5]. Early and effective cognitive training can significantly delay or even reverse cognitive damage, with a reversal rate of up to 26% [6]. This stage is currently the optimal and critical window for cognitive intervention to prevent dementia [7,8].

In recent years, digital cognitive training (DCT), as an emerging training program, has proven more effective in enhancing cognitive functions than traditional paper-and-pencil training [9]. DCT provides personalized training in different cognitive domains based on the performance of older adults, breaking through temporal and spatial constraints. With its convenience, cost-effectiveness, and low operator requirements, DCT is increasingly becoming a preferred alternative [10-12]. In the United States, after the US Food and Drug Administration launched a digital software precertification program in 2017, digital products for cognitive behavioral therapy have been approved [13]. Meanwhile, in China, training software for cognitive dysfunction was approved for the market in 2021 [14], further propelling the development of DCT as a cognitive therapy tool. Millions of older adults have used DCT at home [15], and through digital brain training, they can not only effectively improve cognitive function [16,17] but also reduce the long-term risk of dementia [18].

DCT has shown potential benefits in maintaining cognitive function among older adults and has been applied to prevent and delay cognitive decline in patients with MCI due to its advantages of ease of operation and low cost [19]. Regrettably, significant challenges, such as user attrition, low training completion rates, and weak intentions to continue use, persistently impede its widespread adoption. Over 25% of users reduce participation frequency or drop out of training altogether [20,21], and a striking 52.8% fail to meet the prescribed dosage of cognitive training compliance [22]. After a period of use, compliance and retention rates among some older adults gradually decline [23], with less than 30% of participants completing more than half of the training content [21,24]. Even if older adults with MCI have a positive experience after their initial use of DCT, the intention to continue using it in the future diminishes significantly [25], and they do not continue to use it after the intervention ends [26,27]. More critically, the notable benefit enhancements of DCT largely depend on the long-term continuous use of the training by older adults. Noncompliant behavior not only undermines the potential training effects of DCT [28] but also leads to substantial wastage of medical resources.

Addressing this issue, previous research [29,30] has primarily focused on analyzing the factors that influence older adults’ use or nonuse of DCT after an intervention, treating it as an instantaneous, one-off action. Consequently, low DCT utilization rates are often attributed to older adults’ anxiety about using technology [29], inadequate computer experience [29,31,32], low self-efficacy regarding digital technology [31,32], and inevitable physical decline [33]. Although some scholars have explored the factors affecting the adoption of DCT from the perspectives of health care providers and family members, the explanations have largely been from a structural societal perspective, clarifying why there is severe noncompliance with DCT in practice, such as nursing staff shortage [34,35], inadequate senior management support and attention [34], caregivers’ lack of game technology skills [34,35], and insufficient family supervision [22,36]. These studies have to some extent elucidated the difficulties and barriers encountered in the dissemination of DCT, yet they have overlooked the fact that DCT usage is not a static one-time action but a continuous dynamic process. Moreover, they have not considered the perspectives of older adults with MCI and have not interpreted the behavior of older adults with MCI using DCT throughout the intervention and within their social environment in a phased manner. Within the scope of our literature review, there are currently limited studies on the phases of nonuse and continued use of digital technologies by older adults [26,37]. The troubles and negative impacts of DCT at different stages of use on older adults with MCI, as well as the intrinsic reasons for transitioning from initial acceptance to discontinuous use, have not been adequately explored.

Therefore, from the perspective of this study, continuous and effective usage is key to the successful implementation of DCT programs. We adopted the extended model of IT continuance (ECM-ITC) and conducted qualitative interviews to better understand the reasons behind the shift from acceptance to discontinuous usage among older adults with MCI at different time points before and after using DCT. This model is a representative theoretical framework for exploring individuals’ continuance intentions and behaviors [38], providing in-depth insights into the formation processes and motivations of continuous usage behavior in the digital technology domain [39]. Continuance intention, a critical driver of long-term technology use, depends on users’ perceived usefulness of the system and their satisfaction with its use, both of which are influenced by the degree of expectation confirmation. Consequently, this research employs the ECM-ITC to conceptualize the usage of DCT by the MCI population as a dynamic and continuous process. The objective is to understand the reasons contributing to the phenomenon of discontinuous usage over time during the training process and to delve into the perceptions and actual needs of older adults with MCI and their families regarding DCT usage. By starting from their real-life contexts, we explain the reasons for high attrition rates during the continued use phase and provide decision support to enhance the adoption of intervention strategies.

## Methods

### Study Design

This was a convergent parallel mixed-methods study in which 2 research teams concurrently collected quantitative and qualitative data. The analysis findings from both methods were integrated when interpreting the results to obtain a more comprehensive understanding of the study results [40]. However, this study focuses solely on qualitative data, aiming to deeply explore the underlying reasons that affect the discontinuous use of DCT from the perspectives of older adults with MCI and their families. For the quantitative aspect, we assessed the effectiveness of DCT through an intervention program involving three 30-minute sessions weekly over a 1-month period. The DCT tool used in this study is a cognitive intervention platform based on a gamified design that aims to improve users’ cognitive functions by promoting training in various cognitive domains. This tool covers multiple cognitive areas, including memory, attention, logical reasoning, perception, processing, and executive functions. The DCT tool is accessed through WeChat Mini Programs, the most widely used mobile app in China. Its main features include artificial intelligence chat, gamified training (16 training games with 90 levels each; continuously updated), and progress feedback tracking, providing interactive and timely feedback. Additionally, the gamified training is designed with detailed usage instructions and progressive difficulty adjustments to continuously stimulate cognitive improvement. The qualitative component of our study was based on the ECM-ITC, which provided the framework for developing interview outlines and for organizing and analyzing data [38]. This model serves as a representative theoretical framework that explores the processes and motivations behind individuals’ continued use of digital technology [39]. This study conducted semistructured qualitative interviews using an interview guide before and after using DCT. This qualitative research complied with the COREQ (Consolidated Criteria for Reporting Qualitative Research) guidelines [41].

### Patient Recruitment

We employed a purposive sampling method to select participants who satisfied the inclusion criteria and could provide rich information. Eligible older adults with MCI and their family members participated in cognitive screening and DCT at a community health service center in Guangzhou, Guangdong Province. The inclusion and exclusion criteria for both the qualitative and quantitative components of the study were identical. The inclusion criteria for patients were as follows: (1) aged 60 years or older; (2) met the diagnostic criteria for MCI (subjective cognitive decline, such as memory loss, reported by the patient or family member; objective evidence of cognitive decline confirmed using the Mini-Mental State Examination [MMSE] screening tool [cognitive impairment cutoff scores set according to different educational levels [42]: illiterate, <20 points; elementary school, <23 points; secondary school or above, <27 points]; further assessment of participants who tested positive on the MMSE and confirmation by professional geriatricians or neurologists based on the 11th revision of the International Classification of Diseases [ICD-11] [43] and Petersen criteria [44]; essentially normal daily living abilities, although complex instrumental activities of daily living might show slight impairment; and did not fulfill the diagnostic criteria for dementia); (3) possessed a smartphone and was able to use WeChat; and (4) provided informed consent (participation was voluntary). The exclusion criteria were as follows: (1) concurrent other neuropsychiatric disorders, such as epilepsy, stroke, and Parkinson disease; (2) severe cardiovascular or cerebrovascular diseases, malignant tumors, or other major physical illnesses; (3) significant sensory-perceptual disturbances that could affect assessment or training; (4) severe visual or auditory impairments hindering communication; and (5) participation in any other cognitive training studies within the past 6 months. The inclusion criteria for family members were as follows: (1) aged 18 years or older, with clear awareness and the ability to read, understand, and express in Chinese normally; (2) had familiarity with the patient’s condition, was involved in daily care, and was capable of decision-making; and (3) provided informed consent (participation was voluntary). The exclusion criterion for family members was a history of severe mental illness or physical disease. Health care providers at the community health service center conducted cross-assessments using both paper-based and digital cognitive screening tools. After review by a psychiatrist, 21 older adults met the inclusion criteria. Subsequently, researchers, with the assistance of the community health service center staff, recruited patients with MCI and their family members through on-site face-to-face or telephone interactions. Ultimately, 19 older adults with MCI and 4 family members agreed to participate in DCT. Two older adults declined to participate due to blurred vision or lack of interest.

### Data Collection

Data for this study were collected between January and March 2024, encompassing a total of 38 interviews. We conducted semistructured one-on-one interviews with 19 older adults with MCI and 4 family members both before and after the 1-month intervention period. During the interviews, the older adults and their family members were interviewed independently, each in separate rooms. The interview guide used the same version for all participants, with consistent questions. However, the phrasing of the questions was appropriately adjusted according to whether the respondent was an older adult or a family member, ensuring that the questions were more aligned with their context and level of understanding. The average duration of the interviews, both before and after use, was 21 minutes (ranging from 12 to 80 minutes). The interview questions were formulated based on a literature review and the ECM-ITC framework and were further refined following discussions with the research team, as well as consultations with experts in cognitive impairment screening and management, and qualitative research specialists, culminating in the finalized interview guide (Multimedia Appendix 1).

At their first offline participation in DCT, patients and their family members who met the inclusion criteria were invited to join a WeChat group managed by health care providers, social workers, and researchers for cognitive training. After setting up the DCT at the end of the interview, we assisted the older adults and their families in accessing the Thoven mini-program training games via a WeChat QR code. We guided them on-site on how to open the WeChat mini-program, operate the training games within the mini-program, interpret the game rules, and check their personal training scores. Finally, we reconfirmed that the participants had learned all the training steps and reiterated the prescribed dosage of the training (3 times a week, 30 minutes each session, for a duration of 1 month). Subsequently, during the DCT process, participants received WeChat group messages from staff twice a week. These messages included rankings of the week’s training completion status, encouragement to complete DCT, and real-time answers to older adults’ questions to help them persist with the training.

During the first interview (after enrollment but before intervention commencement), 14 of the 19 participants underwent face-to-face interviews in community health service center meeting rooms, clinics, or community senior activity centers. The remaining 5 participants, due to scheduling conflicts, opted for telephone interviews at a convenient time after returning home. The preuse interview questions primarily focused on their perceptions of cognitive decline, expectations for improving cognitive health, and willingness to engage in DCT.

After the 1 month of training, all participants were invited for second interviews (postuse interview). Of the 19 participants, 15 engaged in face-to-face interviews in community health service center meeting rooms, clinics, or community senior activity centers. The remaining 4 participants, due to scheduling conflicts, opted for telephone interviews at a convenient time after returning home. The older adults with MCI and their family members shared their experiences with DCT, while researchers assisted in reviewing their preuse expectations and motivations, and discussed the extent to which actual experiences matched initial expectations, the perceived usefulness of DCT, reasons for either persisting with or dropping it halfway through the process without continuing to use it, and their intentions to continue using it in the future.

All interviews were conducted by 2 interviewers (authors SZ and CC), both of whom had received training in qualitative research and had experience conducting qualitative interviews. All interviews were carried out in Chinese and were audio-recorded with the consent of the older adults and their family members prior to the start of the interviews. The recordings were transcribed verbatim within 2 weeks following the interview. During the interview process, researchers also observed and collected nonverbal data, such as the participants’ facial expressions, body language, and emotional changes. Before commencing the second interview, researchers reviewed transcription data from the participants’ previous time points to aid in recalling their participation expectations and motivations. Data collection ceased when no new information could be extracted from the analysis of the new interview data, which was achieved after 34 interviews, signifying data saturation.

### Data Analysis

Data collection and analysis were conducted concurrently. Deductive thematic analysis was employed to analyze the individual interview data [45]. The coding was carried out using NVivo 12.0 software (Lumivero), and manual coding was performed in Microsoft Excel. The entire analysis process was completed under the guidance of NG and JL. The ECM-ITC was used as the coding framework for categorizing subthemes. First, the first (SZ) and second (MW) authors transcribed the original recordings into text and familiarized themselves with the content through repeated readings. Throughout the analysis, initial codes were independently assigned by repeatedly reading the transcripts, listening to the recordings, and incorporating field notes. These codes were then organized and categorized into corresponding subthemes. Subsequently, the research team, together with NG and JL, reviewed and discussed these codes and subthemes. Next, the first author (SZ), under the guidance of NG, who has extensive qualitative research experience, applied a deductive framework analysis method to construct a thematic matrix in Microsoft Excel. This matrix displayed themes, subthemes, and quotations, mapping the subthemes onto the components of the ECM-ITC framework (expectation confirmation, perceived usefulness, and continuance intention). Finally, the first author and all research team members collectively reviewed and discussed the themes and subthemes until a consensus was reached.

### Ethical Considerations

This study meets the institutional requirements for human trials and has been approved by the Biomedical Research Ethics Committee of the School of Public Health, Sun Yat-sen University, China (ethics approval number: 2023-129). Subjects have voluntarily signed informed consent forms. Prior to the interviews, participants received both verbal and written explanations about the study, including its objectives, its methods, the approximate duration and frequency of the interviews, and other details. All participants signed written informed consent forms and were given the right to withdraw from the study at any stage. Data from all participants and institutions have been anonymized. Moreover, this research followed the ethical principles of the Declaration of Helsinki.

### Rigor

To ensure that the research findings authentically reflect the perspectives of the participants, we scrutinized and assured the rigor of the data based on the criteria established by Lincoln et al [46], which include confirmability, credibility, transferability, and dependability. The purposive sampling method was employed based on the research objectives and questions to select participants, ensuring the complexity and relevance of the subjects under study, thereby yielding a rich diversity of insights. In addition, the credibility of the research was enhanced by multiple rounds of qualitative data collection. Throughout the study, field notes and memos were used, and a thorough description of the participants and the data collection process was provided to ensure transferability and dependability. Additionally, the study followed a consistent process and employed the ECM-ITC model framework to guide the design of the interview guides, as well as data collection and analysis, thereby enhancing its dependability. Confirmability was established through the systematic handling of recordings and interview transcripts by 2 researchers and the collaborative discussion of data coding among the members of the research team.

## Results

### Overview

This study conducted a total of 38 interviews with 19 older adults with MCI and 4 of their family members, both before and 1 month after implementing DCT. The sample size was determined based on the principle of informational saturation. Data saturation was achieved after interviewing 17 participants, and we conducted 2 additional interviews to confirm that no new information emerged from the new data. Interviews were carried out either at the participants’ homes or on-site. Due to the inability of some participants to attend in person, 9 interviews were conducted via telephone, while 29 were conducted face-to-face on-site. The mean age of the older adults with MCI was 73.3 years (range 60-90 years). Their characteristics are presented in Table 1.

**Figure 1:**
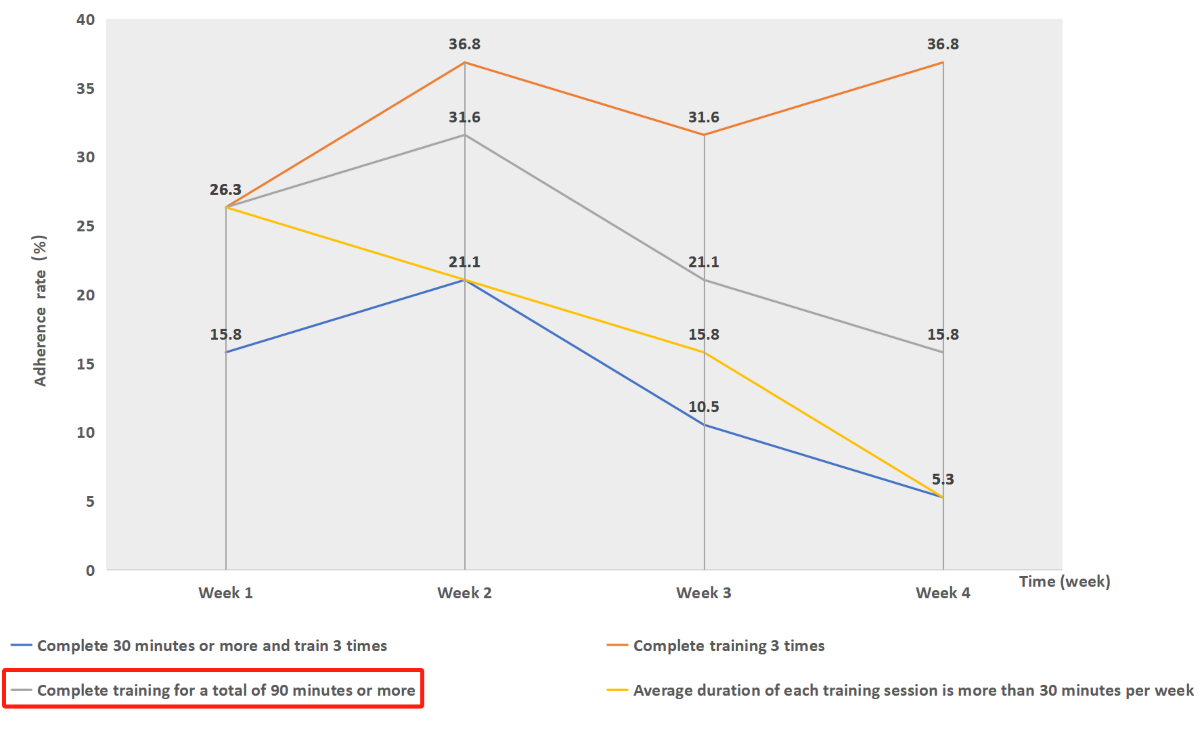
Adherence rates of digital cognitive training.

Among the 19 participants, only 32% (6/19) completed the recommended 90 minutes of weekly training, while 68% (13/19) of respondents used DCT occasionally or even discontinued its use. Detailed variations in the participants’ training over the 1-month period are illustrated in Figure 1.

**Figure 2:**
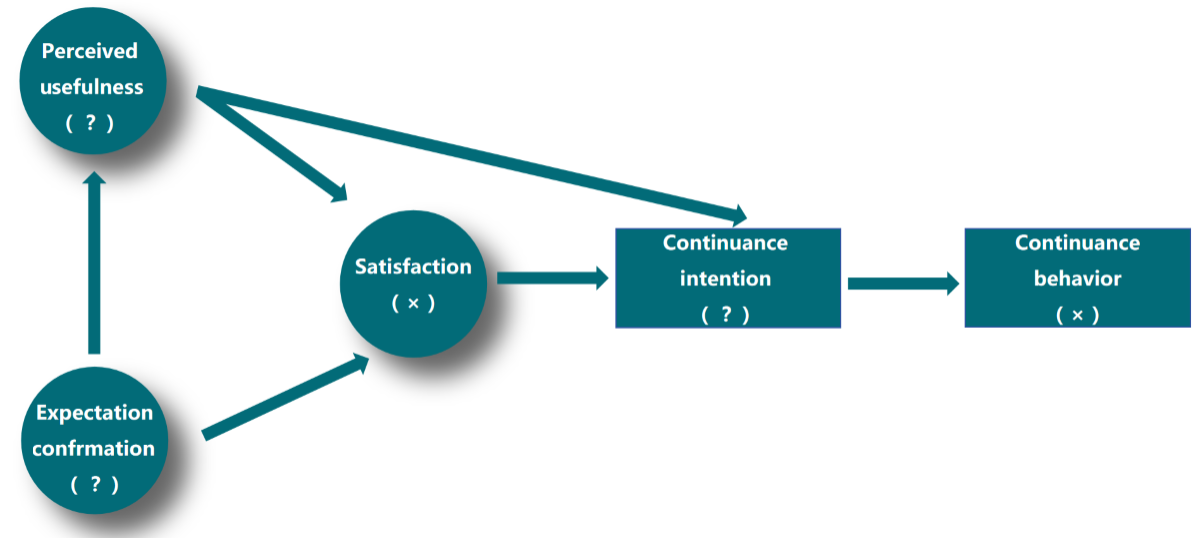
Extended model of IT continuance in digital cognitive training.

In this study, we analyzed the results based on the ECM-ITC (Figure 2) and identified 3 primary themes that revealed the reasons behind the discontinuous usage behavior of DCT among older adults with MCI: (1) discrepancy between vague expectations and actual training experience; (2) unclear perceived usefulness; and (3) suppressed intention to continue usage.

**Table 1:** Participant characteristics.

Characteristic			Value
**Persons living with mild cognitive impairment (n=19)**			
	**Age (years)**		
		Mean (SD)	73.3 (7.2)
		Range	60-90
	**Sex, n (%)**		
		Male	6 (32)
		Female	13 (68)
	**Highest level of education, n (%)**		
		Elementary school	4 (21)
		High school	11 (57)
		College or trade school	4 (21)
	**Living arrangement, n (%)**		
		Staying alone	6 (32)
		Staying with spouse only	6 (32)
		Staying with family members	6 (32)
		Staying with helper	1 (5)
	**Medical history, n (%)**		
		Hypertension	7 (37)
		Diabetes	7 (37)
**Family members (n=4)**			
	**Relationship with the patient, n (%)**		
		Daughter	2 (50)
		Wife	2 (50)

#### Theme 1: Discrepancy Between Vague Expectations and Actual Training Experience

To comprehend the needs of older adults with MCI for DCT, it is crucial to consider the cognitive background and social context in which they live. The majority of participants (14/19, 74%) generally had limited awareness of cognitive impairment diseases and were influenced by misconceptions and prejudices of others regarding cognitive disorders or dementia in their social environment. This led to older adults with MCI needing immense courage to confront discrimination and fear. Cognitive biases and the stigma associated with the disease created confusion in their decision-making process on how to improve memory, causing them to embark on DCT with a sense of bewilderment. However, negative training experiences after use made patients with MCI and their families realize that DCT does not align with their needs. This implies that a greater gap between the ambiguous expectations before using DCT and the actual experience thereafter is associated with a lower level of expectation confirmation. Consequently, over half of the participants (10/19, 53%) expressed reluctance to continue using DCT.

##### Cognitive Bias and Stigma of Cognitive Impairment

Regarding the issue of memory decline, older adults often held misconceptions. Most respondents (11/19, 58%) regarded memory deterioration as a normal phenomenon of “aging” rather than a manifestation of disease, thereby failing to fully comprehend the dangers posed by the cognitive impairment:

Nowadays, I see that 80% of older adults are forgetful and the brains have degenerated. It’s just the way it is when you’re old.

Participant #14, female

Beyond the barrier of insufficient disease awareness, younger older adults were highly confident in their brain health, firmly believing that the usage of DCT is exclusive to dementia patients and incongruent with their current age stage. A family member of an older adult with MCI who had given up DCT usage expressed that younger older adults are reluctant to acknowledge their aging because they consider themselves as “still young” and not yet at the stage where they need cognitive training.

They think it’s useless, they’re still young, they don’t acknowledge that thing (DCT), they feel it’s not yet time to engage with it.

Family member #4, female

In contrast, older seniors often lacked confidence in using DCT. Due to age-related and physical limitations, they were unable to master the use of smartphones and DCT. Older adults with MCI tended to adopt a go-with-the-flow attitude toward their future lives, which to some extent reflects a negative correlation between advanced age and adherence to DCT. One family member, when talking about cognitive improvement through training, frowned, sighed, and indicated that a sense of meaninglessness in life arises in old age.

It’s not that I don’t want him to do the training, but he (older adults) doesn’t care about anything, has completely withdrawn from society, and never trying to get better either, never wanting to change.

Family member #1, female

Prejudices against cognitive impairment from those around them made it difficult for older adults to accept cognitive decline and engage with DCT. In the context of this environment, facing cognitive training was particularly challenging. Some participants (2/19, 11%) often chose to hide their condition and remain silent when confronted with cognitive impairment-related diseases.

Yes, I have memory decline, but I dare not speak up face to face.

Participant #16, female

##### Disorientation and Perplexity Regarding Cognitive Training: Not Knowing What They Want

When older adults with MCI were informed of their cognitive decline, both patients and their family members often found themselves at a loss. Respondents expressed a need for cognitive improvement when asked about methods they believe could enhance memory. However, they were unclear about which type of cognitive training they desired and were even confused about whether effective treatment methods existed.

I want to improve my memory, but I don't know how to do it. Taking medication doesn't seem very convenient either.

Participant #16, female

I don't know (what can improve memory) anymore, maybe participate in activities.

Participant #18, female

Is there a way to improve (memory)? Does such a thing exist? (asked with confusion and urgency)

Family member #2, female

##### Negative Experiences With Training Games: Knowing What They Do Not Want

Negative experiences during the training process can hinder older adults with MCI from actively trying again, reducing their interest and motivation in training games. Some participants (3/19, 16%) pointed out a disconnect between the design of certain training games and the preferences of older adults, stating that the content was “too juvenile” and failed to adequately meet the actual needs of older adults. Others felt frustrated by the training challenges they encountered. After these negative experiences, older adults with MCI and their family members were clear that they did not wish to continue using DCT in the form of training games. In their logic, some traditional training activities (such as playing mahjong, reciting textbooks, etc) could achieve the same effect, leading to a decreased intention to continue using DCT:

I didn’t play Whac-A-Mole; it's not fun, feels like something for kindergarten kids.

Participant #13, female

I’m really not interested in this game; I've already deleted it. Simpler would be better; this is too complicated.

Participant #5, female

Playing this (game), I want to delete it because it's just testing my memory, I might as well recite a book instead.

Participant #19, female

These little games are no good, Playing this kind of thing isn’t as good as playing other things, let him play cards or mahjong tomorrow.

Family member #3, female

#### Theme 2: Unclear Perceived Usefulness

The majority of participants (11/19, 58%) revealed a preference for on-site group training over at-home DCT. Older adults with MCI generally tended to favor what they perceived as substitutable training methods. The perceived usefulness was influenced by the extent of expectation confirmation. A greater discrepancy between preuse expectations and actual postuse experiences was associated with greater skepticism about the usefulness of DCT among older adults with MCI. Although the interviewees acknowledged that persistent long-term training might improve cognitive abilities, the delayed gratification characteristic of DCT did not meet the urgent need for immediate effect among older adults with MCI. This unmet need for instant feedback can influence their attitude toward the usefulness of DCT, which remains uncertain.

##### Preferences for On-Site Training and Substitutable Methods

Most older adults (9/19, 47%), especially those living alone, disliked loneliness and desired “someone to interact and communicate with” to alleviate loneliness. Therefore, on-site group training aligned better with their daily habits and helped establish an emotional support network.

I think it should be offline. I did it with her and felt that we still need to have some of this companionable atmosphere. She keeps asking me, ‘Am I the only one? Are there other people?’ It’s much better with companions around.

Family member #4, female

Out of concern for being socially isolated, many participants (7/19, 37%) mentioned that they cultivated a variety of other interests and hobbies to enrich their lives and maintain social connections.

For her, she might be more interested in things like going for a walk by the river, dancing, and practicing Tai Chi. Given a choice, she would choose (other activities). Playing games would definitely be her last choice.

Family member #4, female

Actually, I have quite a broad range of interests. I like singing, (and) some reading aloud. Sometimes, I also play Idiom Fill in the Blanks on my mobile phone. (As she speaks, she takes out her phone to show photos of herself singing)

Participant #9, female

##### The Conflict Between “Delayed Gratification” of Digital Cognitive Training and “Immediate Gratification” Among Older Adults

The management of cognitive impairment diseases and the improvement of cognitive outcomes rely on sustained long-term training. However, for older adults with MCI undergoing training, immediate positive feedback on memory enhancement was crucial. They believed that achieving “immediate gratification” is key to ensuring adherence to training. One participant expressed a desire for immediate results following training.

I wish it would work right after one day of training, the sooner the better.

Participant #13, female

However, the greatest challenge of DCT was “delayed gratification,” because the effects of the training could not be perceived in time. More than half of the patients (13/19, 68%) found it difficult to continue training when they could not see short-term progress, ultimately choosing to “lie flat” or even “give up.”

If it’s effective, we’ll keep doing it; if not, we won’t do it (shaking head). A little progress in a week is fine. If there’s no effect, spending time on it is a waste.

Participant #5, female

I know (it requires training over a period of time to be effective), just like the health care products I’m taking now. If trying them out shows some effect, I’ll continue buying them; if they haven’t effect, then I won’t buy them anymore. It’s like that.

Participant #17, female

##### Uncertainty About the Usefulness of Training

Older adults with MCI often reduced or withdrew from training before achieving their cognitive enhancement goals, which prevented them from timely experiencing the positive changes that the training could bring and left them with limited knowledge of the importance and potential benefits of DCT. One participant viewed DCT as a tool to provide recreational value and lacked confidence in it as an intervention that could effectively improve cognitive function.

I don’t know if it’s useful or not, I just play with it.

Participant #17, female

Interestingly, even those older adults with MCI who had higher compliance with DCT training expressed concerns about whether brain game training can genuinely delay cognitive deterioration, further exacerbating the skepticism among older adults with MCI regarding the usefulness of the training.

It should be useful after training, it’s bound to be somewhat better.

Participant #12, male

It (Memory) is all more or less the same, learning a bit more might make the mind a bit more enlightened, but it’s hard to say whether it will prevent dementia.

Participant #18, female

#### Theme 3: Suppressed Intention to Continue Usage

The intention of older adults with MCI to continue using DCT was not only influenced by their perceived usefulness but also constrained by various external driving factors. Concerns about the economic risks associated with DCT, lack of support from family members, stringent task requirements from intervention personnel, and the absence of incentives were among the reasons that could trigger their dissatisfaction or even resistance. These external factors negatively impacted the acceptance and user experience of DCT, while participant satisfaction with training was a crucial determinant of their continuance intention. Participant dissatisfaction can weaken their intention to use DCT to delay cognitive decline, even if the training itself is beneficial to cognitive function.

##### Concerns About the Economic Risks of Game Training

Older adults with MCI generally lacked trust in DCT’s QR scanning behavior and training game systems. This low level of trust often stems from negative experiences they have heard from others. Participants expressed concerns that the QR codes might not be a reliable source and that the services and content accessed through scanning could carry the risk of online fraud. One participant, despite being willing to scan QR codes, remained cautious and stated:

At that time, when I entered the mini-program, I was also really scared. There are many scams targeting older adults. It would be really bad luck to encounter one, especially now with so much online fraud.

Participant #14, female

Additionally, this distrust also led some participants to be highly skeptical about the motives behind data collection by the community health service center staff and those conducting the intervention, worrying about potential breaches of personal information privacy. This made them hesitant to engage in any activities involving QR scanning.

You don’t know what might happen after scanning QR codes. I’m afraid of being charged or that they might do something to the phone after scanning, so I don't like scanning QR codes.

Participant #17, female

Among older adults with MCI, even when explicitly informed that DCT was free to use, respondents still worried about potential hidden fees and additional expenses during the DCT process.

That’s why I’m afraid to play the games; I’m worried about charges, concerned about deductions from my phone bill.

Participant #16, female

##### Avoidance of Cognitive Training Under the Pressure of Family Relationships

The pressures associated with family relationships also had a negative impact on participation in DCT. When discussing the role of their families in supporting and encouraging them to persist with DCT, participants expressed reluctance to proactively inform their family members about their involvement in DCT. They believed that they would not receive understanding or attention from their family members. Additionally, due to heavy family responsibilities and pressures, compared to the misfortunes of their lives, memory decline was rather not a bad thing for them. As a result, engaging in cognitive function training was merely an optional aspect of their lives. An older adult, who had long been responsible for the entire household’s chores, mentioned that her life was consumed by a myriad of family tasks, leaving her with almost no personal rest time, let alone time to use DCT during her leisure.

My son doesn’t care about me at all. It’s better not to provoke him. My daughter-in-law has the same attitude; she’s very aggressive in the way she speaks. They (my son and daughter-in-law) don’t do any housework; I do everything in the house. We, the unpaid nannies, are all the same. That’s just how it is for older adults in China. So, I’m actually happier during the New Year when no one bothers me.

Participant #13, female

During the training process, understanding and support from family members could help to inspire older adults with MCI to use DCT with enthusiasm. However, participants reported that children rarely took the initiative to care about the health condition of older adults, and complex family relationships could leave older adults with MCI feeling more depressed and demotivated. Sometimes, they felt ignored and lacked the motivation to improve their health. In such circumstances, avoiding DCT became a way for older adults with MCI to alleviate their psychological burden.

My son seldom keeps me company (silent, sighs softly). Even when I was sick and hospitalized, he didn’t even say a word of greeting when he returned home.

Participant #16, female

I got divorced from my spouse, and later, when my child was in college, he got swindled by online gambling. I had to send my child to detention for a month. Sometimes, I just can’t control him. He (my son) only cares about money; you’d better give money to him. Under these circumstances, it’s impossible to feel happy.

Participant #12, male

##### The Intrinsic Paradox of Incentive Mechanisms

Despite older adults with MCI overcoming financial and privacy concerns and being able to obtain support from family and society, they still participated in training in a task-driven, passive manner. Their willingness to persist with the training was primarily a response to the requirements set by community health service centers and their staff. On the one hand, these older adults maintained positive relationships and interactions with the staff at community health service centers or social work stations, which made them more willing to cooperate in activities.

I definitely support you and support your work in completing your studies and careers.

Participant #9, female

On the other hand, older adults with MCI did not have an intrinsic interest in learning about DCT. In this context, the encouragement and strict demands of intervention personnel became significant external forces driving them to passively complete the training tasks.

If you ask me to do it, I’ll get your thing done first thing in the day before doing other things. It’s mainly to finish the task, not because there’s any interest.

Participant #18, female

These older adults viewed the use of DCT mainly as a means to cooperate with staff in completing tasks. Therefore, incentive mechanisms play an important role in prompting them to start or continue using DCT. When asked how to make older adults with MCI more willing to persist with training games over the long term, they expressed a desire for “immediate rewards” during the training process, which could provide a great sense of satisfaction.

To complete the training, you need to post a record of completion in the group to get a red packet. It's like the classes we attend (lectures on cardiovascular and cerebrovascular health), after the class, you post your notes in the group chat, and then every day they give you 8.88 yuan in cash immediately.

Participant #13, female

Furthermore, incentive measures can effectively attract older adults to participate in training in the short term. With rewards available, they treated participating in DCT as a clocking-in task and were thus more willing to continue using DCT and to remember to train regularly.

It’s better with rewards. When there are little perks, they feel like they haven’t done this task today.

Participant #18, female

If there’s a red packet, they’ll remember that time. You just stipulate a time to complete the training and give out the red packet. Some people think about the red packet and then they’re ready to play.

Participant #17, female

## Discussion

### Principal Findings

Our findings indicate that the 3 dimensions of expectation confirmation, perceived usefulness, and continuance intention are critical factors facilitating continuous usage behavior. Older adults with MCI and their families were unclear about the desired form of training they truly wanted prior to using DCT. However, after the actual experience, they explicitly expressed a preference not to undergo interventions through training games. This significant discrepancy between expectations and actual training experience, coupled with the unclarity of the perceived usefulness of DCT, resulted in their low satisfaction with DCT. Over time, various extrinsic factors further suppressed their intention to continue using DCT, making it difficult to establish adherence to DCT and ultimately preventing the formation of continuous usage behavior.

This study employed the ECM-ITC as a guiding framework, specifically focusing on the social environment and daily life contexts of older adults with MCI. It qualitatively explored the critical reasons that influence the shift from initial acceptance to discontinuous usage of DCT throughout the process of the training intervention. The adoption of digital technology is a nonlinear progression from initial acceptance to sustained usage at a later stage [37], and this progression is particularly challenging for the specific group of older adults with MCI. They may face greater challenges in accepting and continuously using digital technologies. Within the ECM-ITC, low levels of self-efficacy in using digital tools and insufficient convenience of participation have been identified by numerous studies as moderating factors affecting the long-term adherence of older adults to intervention measures [29,31,47]. Previous research has primarily concentrated on assessing the immediate effects following the conclusion of DCT interventions. Ample evidence suggests that DCT can significantly improve overall cognitive function, enhance quality of life, and alleviate psychological symptoms, such as anxiety, in patients with cognitive impairments [17,23,48]. However, the enhancement of cognitive training effects should not only focus on the instantaneous effects of short-term use but also prioritize the more important long-term sustained effects, which depend on good adherence from participants. Regrettably, older adults with MCI are reluctant to continue using DCT after receiving the intervention. Why is this the case? We found that prior research often regards the acceptance behavior of DCT as a static decision, conflating the initial acceptance with continued usage and only analyzing the reasons for the low usage rates of DCT based on participants’ postuse experiences and satisfaction. Consequently, this study conducted multiple interviews at different stages before and after training to place the behavior of older adults with MCI using DCT in a dynamic context, exploring the key barriers that prevent their adherence to DCT usage.

Expectation confirmation, which represents the extent to which preusage expectations match postusage actual experiences [38], is a significant initial factor affecting the continuous usage behavior of older adults with MCI. A higher degree of expectation confirmation is associated with a greater likelihood that users will perceive the technology as useful and feel satisfied with it [49]. However, if the individual’s expectations are vague and there is a significant gap between the actual experience and expectations, this internal conflict can weaken the user’s intention to continue using the technology [50]. Our study found that older adults often have cognitive misconceptions and biases about cognitive impairment diseases, and the associated stigma leads to insufficient attention to cognitive health, which reduces their acceptance and willingness to continue using DCT. This is consistent with previous research [51,52] showing that most community-dwelling older adults lack a comprehensive and correct understanding of MCI. Furthermore, some demographic factors, such as age and education level, limit the improvement of disease cognition [51], and older adults with lower educational levels are more prone to experiencing digital technology anxiety [53]. During the DCT process, the initial detailed guidance, encouragement, and reminders provided by the staff enhanced the initial confidence of the older adults participating in the training. However, as the difficulty and complexity of the training increased and the content went beyond the scope of the initial guidance, the gap between the frustration of the actual training experience and the expected confidence led to a gradual decline in the older adults’ interest in DCT. This further weakened their motivation and sense of participation. In the social environment of older adults with MCI, their logical thinking tends to perceive traditional entertainment activities, such as playing mahjong, as more compatible with their accepted method of exercising the brain than DCT. For older adults with MCI, this preference stems from the simplicity and familiarity of traditional entertainment activities, which do not require them to learn new skills or adapt to new rules. In addition, these activities not only provide brain training but also enhance manual dexterity and emotional connections.

This study also found that older adults with MCI are caught in a paradox between the need for immediate gratification and the delayed gratification characteristic of DCT. The effects of DCT are often gradual and not immediately perceptible. Evidence suggests that the average duration of digital health interventions spans from 4 to 24 weeks [54], and it takes 5 to 7 weeks of cognitive training for older adults with MCI to exhibit improvements in working memory [55], a perspective supported by our study results. After completing DCT, older adults can receive immediate visual and tactile feedback, such as improved training scores, increased accuracy, and enhanced hand-eye coordination, allowing them to perceive progress in game task completion and improvements in operational abilities. However, improvements in cognitive functions like memory and executive function often take a longer period of time to manifest. Older adults with MCI are unable to perceive substantial memory improvements from the game in the short term, leading to a lack of sufficient confidence in the usefulness and effectiveness of DCT [28]. This lack of confidence leads them to prefer on-site training or training methods with social attributes [56]. From the patients’ perspective, the current research also highlights the importance of combining cognitive training with physical exercise. Previous studies have noted that compared to gamified cognitive training, motor-cognitive training not only has higher ecological validity but also is more effective in improving cognitive function performance [57,58]. In the context of the widespread prevalence of intelligent and digital technologies, older adults frequently hear many promises about uncertain future outcomes during their involvement in digital interventions. For them, immediate experiences and immediate gratification have become important requirements and experiences in their daily lives, driving a preference for activities that offer immediate satisfaction rather than digital intervention programs that require long-term commitment to see results. Therefore, combining exercise science with cognitive training that provides immediate gratification aligns better with the primary preferences of older adults.

Finally, the interviewees shared their suppressed intention to continue usage, which is a key factor directly affecting individuals’ continuous usage behavior. Previous research has shown that a general lack of trust in digital technology and its sources of promotion [59-61], along with significant life stressors [62], hinder the adoption of digital health care by participants. More importantly, usage intentions driven by genuine self-awareness and autonomous motivation to satisfy intrinsic psychological needs are of higher quality, encouraging participants to develop more positive and sustained health behaviors [63]. However, older adults with MCI often choose to participate passively in training, which makes it difficult for them to achieve genuine cognitive improvement from the training. Our study further found that participants hoped to be motivated to persist in using DCT through incentive mechanisms. In China, older adults are considered a “digitally disadvantaged group,” with the internet penetration rate among those over 60 years at just 11.3% [64]. For them, the busy lifestyle habits formed in the past nondigital era have solidified their lifestyles, and any activities beyond their daily routines are seen as an additional burden. Only continuous incentives can stimulate their motivation to engage persistently. This aligns with the principles of the achievement goal theory in the social psychological motivation theory, which suggests that motivations driven by external incentives tend to be performance oriented, with goal completion based on external reward mechanisms. However, fostering a mastery-oriented climate can enhance the sustainability of behaviors [63]. While short-term rewards may encourage older adults to participate in training, if the training is seen as a task rather than an opportunity to improve health, it becomes challenging to cultivate a positive motivation model and sustain participation behavior among older adults, and the effectiveness of such incentives is greatly diminished. Moreover, long-term reliance on material rewards not only increases the burden on operating organizations but also may lead to the inability of older adults to continue using DCT after the withdrawal of rewards.

In summary, the behavior of older adults with MCI in using DCT is not static but evolves dynamically over time with accumulated usage experience. Our research findings highlight that the discrepancy between expectations and reality can undermine the perceived usefulness of technology for patients with MCI, jointly reducing their satisfaction. This negative experience further suppresses their continuance intention, ultimately leading to an inability to sustain long-term usage behavior.

### Implications for Future Research and Practice

Future research should thoroughly understand the expectations of older adults with MCI regarding DCT before developing training programs and should provide cognitive training formats that better meet their needs based on their postusage feedback [28,65], thereby narrowing and bridging the expectation gap. At the same time, older adults with cognitive impairment should be encouraged to gradually achieve the set goals during interventions to build confidence in sustained participation. In the training of older adults with MCI, by fully stimulating both the intrinsic and extrinsic motivational forces of these older adults, the intention to persistently engage in training can be enhanced, thus achieving a more sustained and effective cognitive training effect. Integrating qualitative research into the program development phase to dig deeper into the key variables that impede continuous participation can provide guidance for subsequent studies. This approach can help explore how to effectively facilitate the conditions and implementation pathways for sustained use. Existing research has shown that co-design procedures can not only identify barriers to accessing rehabilitation for people with dementia but also guide the development of potential solutions [66]. Future studies should involve multiple stakeholders, including older adults with MCI, their family members, and health care providers, in the co-design process of DCT programs. This will help researchers better capture and understand the actual needs and real-life use scenarios of users, thereby integrating DCT into the daily lives of older adults.

Given that older adults generally lack a correct understanding of cognitive impairment and hold a nihilistic attitude toward the existence of effective training methods, this cognitive bias further affects their proactive acceptance of DCT. Therefore, future intervention plans should enhance the scientific understanding of cognitive impairment and DCT among older adults through knowledge education and training. These efforts can help older adults form reasonable expectations about the content and format of DCT, thereby narrowing the gap between expectations and actual experiences. To effectively improve cognitive function, researchers should consider combining DCT with offline exercise training. This integrated approach represents a critical pathway for developing optimal interventions in the future. Only then can intervention programs adequately meet older adults’ preferences and needs for DCT and achieve the successful implementation and sustained application of DCT in community or in-home settings.

### Strengths and Limitations

In this qualitative study, we adopted the ECM-ITC for the first time to view DCT usage behavior as a dynamic evolutionary process. This model guided our data collection and analysis, providing a comprehensive explanation for the intrinsic reasons of the behavioral shift from initial acceptance to eventual discontinuous usage of DCT among older adults with MCI across different stages of the preuse and postuse periods. Another advantage of this study is the focus on older adults with MCI, placing their attitudes and behaviors regarding the inability to sustain DCT usage within the context of the entire intervention process and their social living environment. Additionally, incorporating the perspectives of family members provides holistic, family-centered insights.

The study also has some limitations. First, despite our efforts to recruit participants of different genders, over two-thirds of the participants were female, which may be related to the higher prevalence of MCI in women [67]. In addition, we collated and analyzed data based on a predetermined theoretical framework, which might have overlooked important factors outside the ECM-ITC. Finally, although we encouraged family members to participate in the training of older adults with MCI, the sample size of family members was still relatively small. Additionally, the study was conducted in only 1 community health service center in Guangzhou, which may limit the generalizability of the study’s findings. Future research could collect more data at the family level for a comprehensive understanding of this phenomenon.

### Conclusions

Currently, there is extremely limited research on the discontinuous usage phase of DCT. By using the ECM-ITC, this study comprehensively gained insights from older adults with MCI and their families, identifying reasons for the transition from accepting DCT to discontinued use of DCT among adults with MCI aged 60 years or older. There are opportunities to better engage older adults in adhering to DCT in the future, but it is important to consider that cognitive training is only one part of their lives and to recognize the complex needs of older adults as well as the challenges they may face when using DCT. Future research should optimize and integrate these reasons into intervention programs, designing personalized interventions that align more closely with the needs of older adults to ensure that DCT can be genuinely integrated into the daily lives of older adults with MCI.

## Data Availability

For protecting participant rights and preventing the potential identification of participants through the complete interview transcripts, the data generated and analyzed during this study will not be made publicly available. However, upon reasonable request, anonymized format summaries of the interview content analysis and relevant data supporting the research findings can be obtained from the corresponding author.

## Appendix

Multimedia Appendix 1 Interview guide.

## Abbreviations

DCT: digital cognitive training
ECM-ITC: extended model of IT continuance
MCI: mild cognitive impairment
MMSE: Mini-Mental State Examination
