# Supplementary material for: Exploring the Discontinuous Usage Behavior of Digital Cognitive Training Among Older Adults With Mild Cognitive Impairment and Their Family Members: Qualitative Study Using the Extended Model of IT Continuance
Source: J Med Internet Res. 2025 Mar 25;27:e66393. doi: 10.2196/66393 (PMC11979547; doi:10.2196/66393)
Supplement: Multimedia Appendix 1 [file jmir_v27i1e66393_app1.docx]

**Multimedia Appendix 1.** Interview guide.

**Acceptance to participate in digital cognitive training (DCT)**

| **Main question** | **Domain** |
| --- | --- |
| Have you noticed any memory decline in your daily life? Could you describe the performance of your memory decline at that time through some incidents?(eg, grocery shopping, cooking, travelling and some other daily activities); Do you believe that memory decline issues needs to be treated or improved? Why? | Memory Decline |
| Imagine there is now something that can enhance memory, What do you imagine it might be like? | Training Expectations |
| You are willing to participate in this training, so how long do you expect to have effective results after participating or rather your memory stops deteriorating or even shows improvement? | Perceived Usefulness |
| The DCT requires you to train 3 times a week on your smartphone for 30 minutes each time, for one month. Would you be willing to continue playing it? If not: why? What kind of training method would you prefer? | Continuance Intention |
| This training requires you to stick to it for one month. What additional help or resources do you think you might need during this process? (eg, material rewards, cash incentives, family and social support, etc.) | Factors Influencing continuous usage |

**Adherence to completing weekly DCT of 90 min or more**

| **Main question** | **Domain** |
| --- | --- |
| How do you feel about your memory recently? Are there any other signs of memory decline? (eg, grocery shopping, cooking, travelling and some other daily activities) | Memory Decline |
| How do you feel about doing this training game on your smartphone? | Training experience and feeling |
| Before you used this training game, what did you think something that could improve your memory might be like? Now that you have used it, do you think any differences between this form of training on a smartphone through games and what you had imagined? | Expectation Confrmation |
| Do you think these training games are useful for improving memory or rather Can it enhance the memory of older adults?  If yes: Could you share an example or experience where you found it useful?  If no: Why? What kind of training method do you think could improve memory? | Perceived Usefulness |
| You have been consistently training for a month, what are the reasons for you to stick with the training? | Reasons for Persisting with Training |
| If there is a similar training intervention program (3 times a week, 30 minutes each time, for 3 months), would you be willing to continue participating?  If not: Why? What kind of training method would you prefer? | Continuance Intention |
| What do you think would encourage older adults more willing to continue with the training games for a longer period? What resources or support are needed? (eg, material rewards like gifts, cash incentives, family and social support, etc.)  When our staff first introduced the training game to you and during your use of it, did you have any other concerns or worries? What aspects were you concerned about? (eg, concerns about scams, hidden fees, sales pitches) | Factors Influencing continuous usage |

**Discontinuous usage of DCT**

| **Main question** | **Domain** |
| --- | --- |
| How do you feel about your memory recently? Are there any other signs of memory decline? (eg, grocery shopping, cooking, travelling and some other daily activities) | Memory Decline |
| How do you feel about doing this training game on your smartphone? | Training experience and feeling |
| Before you used this training game, what did you think something that could improve your memory might be like? Now that you have used it, do you think any differences between this form of training on a smartphone through games and what you had imagined? | Expectation Confrmation |
| Do you think these training games are useful for improving memory or rather Can it achieve the effect you desire?  If yes: Could you share an example or experience where you found it useful?  If no: Why? What kind of training method do you think could improve memory? | Perceived Usefulness |
| What are your reasons for not using or only occasionally using the training game on your smartphone? | Reasons for discontinuous usage of DCT |
| If there is a similar training intervention program (3 times a week, 30 minutes each time, for 3 months), would you be willing to continue participating?  If not: Why? What kind of training method would you prefer? | Continuance Intention |
| What resources or support would you need to keep you doing the training games in the long term? (eg, material rewards like gifts, cash incentives, family and social support, etc.)  When our staff first introduced the training game to you and during your use of it, did you have any other concerns or worries? What aspects were you concerned about? (For example: concerns about scams, hidden fees, sales pitches) | Factors Influencing continuous usage |
